# Giving eyespots a shiner: Pharmacologic manipulation of the Io moth wing pattern

**DOI:** 10.12688/f1000research.12258.2

**Published:** 2017-09-26

**Authors:** Andrei Sourakov

**Affiliations:** 1 McGuire Center for Lepidoptera and Biodiversity, Florida Museum of Natural History, University of Florida, Gainesville, FL, 32611, USA

**Keywords:** Eyespots, wing pattern, Lepidoptera, heparin, phenotypic plasticity, melanism, butterflies, moths

## Abstract

Our knowledge of wing pattern formation in Lepidoptera has advanced significantly in recent years due to the careful examination of several groups of butterflies. The eyespot is a prominent feature of Lepidoptera wing pattern, especially in the family Saturniidae. The present study examined how sulfated polysaccharides affected the wing pattern formation of the Io moth, *Automeris io* (Saturniidae).  Prepupae and pupae of this species were subjected to injections of heparin and cold shock. While the cold shock had little to no effect on wing pattern, the aberrations resulting from heparin injections were moderate to profound and depended on the dose and the stage at which injection was made. The changes consisted of expansion of the black ring around the dorsal hindwing eyespots and distortion of discal spots on both dorsal and ventral sides of forewings, suggesting a possible link between genetic controls of these elements. Several different types of scales form the normal color pattern of *Automeris io*, and heparin-induced changes correspond to changes in shape of scales. The resulting aberrations are dubbed ‘Black Eye’ and ‘Comet Eye.’ Other known aberrations of *Automeris io* eyespots are summarized, illustrated, and named.

## Introduction

While our understanding of the mechanisms involved in butterfly wing pattern development has been increasing exponentially in the recent two decades, the work has been largely limited to butterflies such as *Junonia*, *Heliconius*, *Papilio* and *Bicyclus*. Thanks to these ‘model’ genera, we now understand homologies among wing pattern elements and the adaptive radiation that led to the kaleidoscope of intriguing ‘designs’ found among ca. 160,000 Lepidoptera species ( Martin & Reed, 2010).

Natural and artificially generated aberrations serve as windows into the developmental mechanisms and evolutionary history of animals. In addition to many naturally occurring melanic aberrations and some melanic recessive phenotypes that can be obtained and/or maintained through inbreeding, the dark markings of Lepidoptera wings can sometimes be amplified by the timely application of a colder regime to the immature stages (e.g., Sourakov, 2015 and references within). Serfas & Carroll (2005) first demonstrated that injections of heparin into the early pupal stage can simulate cold shock and alter wing patterns in similar ways. Martin & Reed (2014) utilized heparin injections to understand genetic controls and homologies among separate wing pattern elements.

Eyespots are characteristic of many Lepidoptera, and considerable advances have been made towards understanding their evolution ( Monteiro *et al.*, 2006). In *Automeris io*, a species whose name, if anglicized (‘Eye’‘Oh’!), invokes associations with its pair of magnificent dorsal hindwing eyespots that are exposed when the moth (otherwise cryptic) is threatened. Several recessive mutations causing deformations of the black ring surrounding the dark blue eyespot with a white center have been obtained through inbreeding, conducted first by Thomas Manley (1978, 1990) and, more recently, myself ( Table 1 below). However, the most dramatic aberration, which involves the melanization of almost the entire hindwing, was found in an *A. io* male collected in 1966. It was noticed only recently while the MGCL Saturniidae collection was being re-curated ( Covell, 2012).

**Table 1.** Aberrations of dorsal hindwing eyespots found in *Automeris io*.

Aberration name	Description	Author, details
“Black eye”	Figure 1A and Figure 2D – expansion of black eyespot ring, so that the area between eyespot and outer black band entirely or almost entirely black	Heparin-induced, present study
“Broken eye”	Figures 3B,C – vertical streaks of black medially of the eyespot	Manley, heritable aberration
“Teardrop”	Figure 3D – eyespot shape modified, with an appendix extending towards wing base	Manley, heritable aberration
“Caecus”	Figure 3A and perhaps Figure 4C – eyespot completely disappears, masked by black pigment	Wild, collected by J.L. Boughner; Heparin-induced, present study
“Comet eye”	Figure 1C, Figure 2A – black ring around eyespot with smudges extending towards wing base	Heparin-induced, present study
“Barley eye”	Figure 1E and Figure 3F – black ring uneven, bulging or protruding locally	Cold shock-induced, present study; Obtained through inbreeding by Sourakov
“Winking eye”	Figure 3E – blue circles forming eyespots are of uneven size on left and right wings due to uneven expansion of black ring	Obtained through inbreeding by Sourakov

A previous study by Sourakov (2015), in which the development of the Bella moth was manipulated by temperature change, suggested that the black wing pattern elements of the hindwings may be forming during the prepupal stage. Hence, in the present study, heparin injections were done to both prepupal and pupal stages, and the cold shock was delivered to the former. The results of these injections, while not replicas of known genetic aberrations, are quite dramatic. They are illustrated along with the slight aberrations possibly resulting from cold-shock and heritable aberrations, both those heretofore described and those previously unrecorded.

### Structure of *Automeris io* eyespots (See Supplementary File for figures)

Close examination of both wings in normal individuals reveals not only color but also structural differences between different color pattern elements, supporting similar findings in Nymphalid butterflies ( Iwata & Otaki, 2016). As pointed out by Martin (2017) and Marcus (2017) and is confirmed here ( Supplementary Figure S1) the white center of the small ventral forewing eyespot (DI element) is located on the discal cell’s crossvein between M _2_ and M _3_. Similarly, the dorsal hindwing eyespot center is also tied to the discal cell’s crossvein between M _2_ and M _3_ ( Supplementary Figure S2). Sibling moths can frequently be recognized by the size and shape of the eyespot, the width of each of its elements, and its position on the wing. As the examination of several specimens with differently positioned eyespots suggests, the position of the eyespot on the wing, which in some broods is shifted distally towards the outer black band of the wing, is determined by the wing venation ( Supplementary Figure S2 & Supplementary Figure S3).

Microscopic examination makes it obvious that the white and blue center of the dorsal hindwing eyespot is inset deeper within the wing plane compared to the eyespot’s black ring, which is level with the surrounding yellow areas. Removing successive layers of scales using scotch tape revealed that the ground layer of all three of the above elements is formed by similarly shaped, wide, short cover scales ( Supplementary Figure S3). In that first layer of scales covering the wing membrane, the white (fluorescent in UV light), flat, short, and wide scales, aggregate mostly along the discal cell’s cross vein that runs through the middle of the eyespot. The center of the elongated white spot that these scales form most likely corresponds to the developmental focal point, akin to that of *Junonia coenia,* as described by Nijhout (1980).

The dark-blue similarly shaped, iridescent scales intermingling with additional white scales described above form the blue-and-white area of the eyespot. Their non-iridescent-black and translucent-yellow counterparts also underlie the black ring around the eyespot and the surrounding yellow area, respectively ( Supplementary Figure S3). In the black ring, however, these short and flat ground scales are hidden by another layer of long and flat scales that are ca. 1.5 times longer than the first type and half as wide. These types of scales are absent in the blue-and-white eyespot center ( Supplementary Figure S4). The color of the blue eyespot area can vary depending on the proportion of white scales that is intermixed with the blue ones.

While the scale type that dominates the surface layer of the black ring is also numerous in the surrounding yellow areas, it is not readily visible as it is covered by thin, long, bristle-like scales that are twice as long and only a quarter as wide. As a result, the surface of hindwing outside of the eyespot appears hairy rather than scaly. In the black ring, the bristle-like scales (colored black) are also present, but they are few compared to the rest of the hindwing.

## Methods

Representatives of five broods of *Automeris io* from local stock (over 300 caterpillars) were reared on sugarberry ( *Celtis laevigata*) in Gainesville, Florida, in the fall of 2016, resulting in 130 pupae. Caterpillars were maintained in large clear cellophane bags under a natural light regime in an unheated room with windows. *Automeris io* caterpillars take 2–3 months to develop undergoing 6 (males) or 7 (females) instars. The previously recorded time-lapse photography of the pupation process ( Sourakov & Schlachta, 2016; Sourakov & Schlachta, 2017) allowed for the estimation of age of prepupae and pupae. While the caterpillars were pupating in November, when temperatures fluctuated daily between 10 and 25°C, the pupation process was monitored visually through a slit in a cocoon. It takes ca. 7 days from cocoon spinning to pupation and about 8 hours for a pupa to change color from green to dark brown. Hence, the approximate age of a pupa can be judged relatively easily up to 8 hours after pupation ( Supplementary Figure S5).

Ten pupae of different stages, but not older than 8 hours, were injected using a 10µl syringe with 10µl (1 drop) of heparin solution. The solution was obtained by purchasing 50 mg of heparin sodium salt from porcine, manufactured by MP Biomedicals, Inc. and adding 0.1 ml of distilled water. Additionally, two late-stage prepupae were injected within a day of pupation with 5µl and 10µl of heparin solution. Injections of pupae were conducted under the wing through the soft cuticle separating thorax from abdomen. The two prepupae were injected in the side approximately at the mid-length on the 7 ^th^ day after cocoons were spun and within a day of pupation. Upon injections, prepupae and pupae were placed back into their cocoons and in individual plastic bags with paper towel available for the moth to perch on upon emergence. Additionally, 12 prepupae were subjected to cold shock in the refrigerator (7 C°) for 24 hours. During diapause, all pupae were kept under ambient light conditions, with temperatures fluctuating between ca. 10 and 30°C, until most of the untreated pupae (75%) and some of the injected ones emerged during May-August 2017.

## Results and discussion

**Figure 1.**
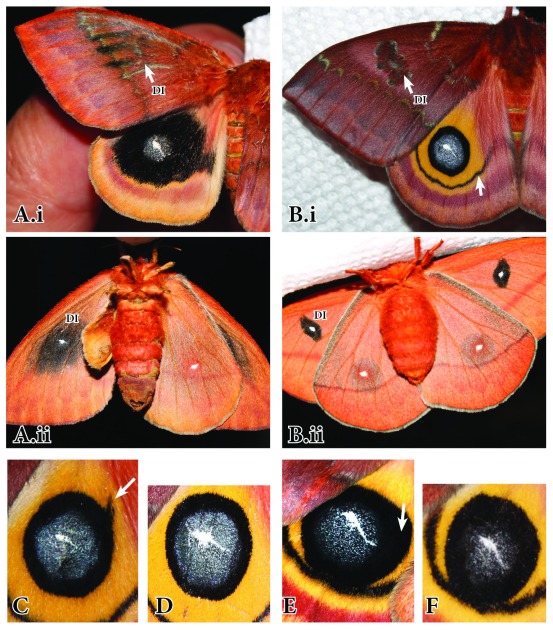
**A. “Black eye” aberration, female.** A female of the Io Moth, *Automeris io*, with wing pattern altered by injection 10µl of heparin solution (sulfated polysaccharide) into the pupal stage (ca. 5 hours after pupation). Voucher FLMNH-MGCL#289216. **B. A normal *A. io* female** from the same brood. (i) dorsal, (ii) ventral surface. **C, E.** Slight aberrations ( **“Comet eye” and “Barley eye”)** of the black ring around eyespots in two *A. io* females cold-shocked as prepupae next to ( **D, E)** control siblings. Photos by Andrei Sourakov.

**Figure 2.**
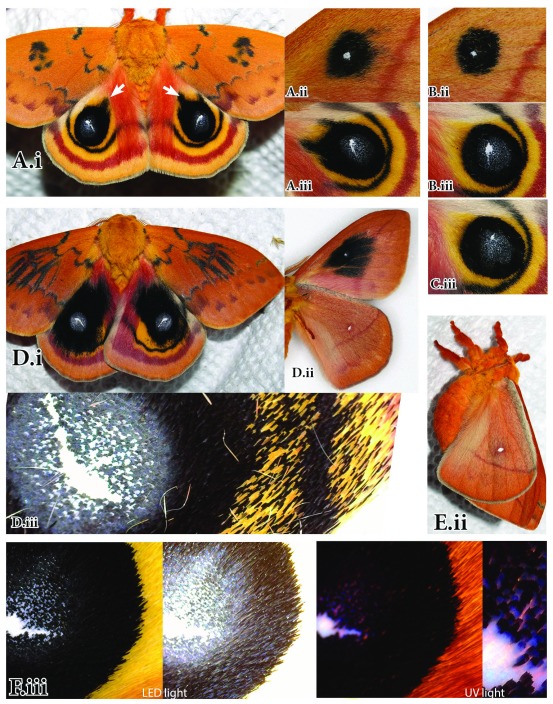
**A. “Comet eye” aberration.** A male of the Io Moth, *Automeris io*, from prepupa injected with 5µl of heparin solution within a day of pupation. FLMNH-MGCL#289217. **B. Eyespots of a normal *A. io* male** from the same brood. Voucher FLMNH-MGCL#289218. **C. Eyespot of *A. io* male** from the same brood whose prepupa was cold-shocked. Voucher FLMNH-MGCL#289221. **D. “Black eye” aberration, male.** A male of *A.io*, with wing pattern altered by injection of 10µl of heparin solution into the prepupal stage within a day of pupation. Voucher FLMNH-MGCL#289220. **E,F. A normal sibling** *A. io* male from control group. (i) Dorsal, (ii) Ventral, (iii) Close-up of dorsal hindwing eyespot. Photos by Andrei Sourakov.

While control pupae and most of the individuals cold-shocked as prepupae had an emergence rate of 75%, and showed little or no deviation from the expected wing pattern, most of the pupae that were injected by heparin did not emerge. The three individuals that emerged (two males from injected prepupae and one female from a pupa injected ca. 5 hours after pupation) exhibited a substantial variation from the norm ( Figure 1 and Figure 2). Additionally, upon close examination and dissection of cocoons and pupae from the rest of the experimental group, five more males were recovered, four of which showed significant deviation from the norm.

A transformed female is illustrated in Figure 1A. Injection may have damaged the right hindwing, so it did not spread properly ( Figure 1A.ii), but the left side was structurally intact and strikingly different, with the hindwing almost entirely black due to the expansion of the black ring around the eyespot. The control sibling female is illustrated in Figure 1B for comparison. Also, in Figures 1C and 1E, the slight changes in the black ring around the eyespot exhibited by two females cold-shocked as prepupae are illustrated next to control siblings.

**Figure 3.**
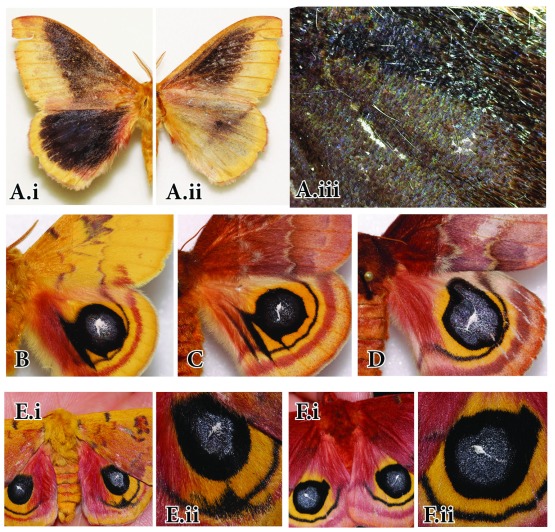
**A.“Caecus” aberration.** A wild-collected *Automeris io* male exhibiting a unique melanic aberration in which most wing pattern elements are obscured by melanin. (i) Dorsal side, (ii) Ventral side, (iii) Close-up of the eyespot area. Collected in Wascott Township, Douglas Co., Wisconsin, on 24 June 1966 by J.L. Boughner; Voucher FLMNH-MGCL#1000907. **B, C. “Broken eye” aberrations** resulting from recessive mutation reared by Thomas R. Manley (1978, 1990). ( **B**) Male 67#3 resulting from cross of wild “broken eye” female crossed with sibling non-aberrant male; Collection of Peabody Museum; YPM No. 843761; ( **C**) Female 67#1 sibling with the above male; YPM No. 843765. **D. “Teardrop” aberration** resulting from a recessive mutation reared by Thomas R. Manley. Cross 13-86 (1986) of normal “teardrop” brood male 10-85 with sib “teardrop” female 27-85. Collection of Peabody Museum, YPM No. 843755. **E. “Winking eye” aberration** resulting from a recessive mutation (expressed through 2 ^nd^ consecutive full-sib crossing) reared by A. Sourakov in 2014. **F. “Barley eye” aberration** resulting from a recessive mutation (expressed through full-sib crossing), reared by A. Sourakov in 2014. Voucher FLMNH-MGCL #166627. Photos by Andrei Sourakov.

Two aberrant males, whose prepupae were injected with 5µl and 10µl of heparin within a day of pupation are illustrated in Figure 2. The one that received a smaller dose was only slightly aberrant in its dorsal hindwing eyespots ( Figure 2A). There, the black rings have smudges extending towards the wing base akin to comet tails, hence the aberration is nicknamed “Comet Eye,” following the tradition started by Manley (1978, 1990), who gave genetic aberrations of *Automeris io* names, such as “Broken eye,” ( Figures 3B and 3C) and “Teardrop” ( Figure 3D).

The male from prepupa that was injected with 10µl of heparin was transformed much more drastically ( Figure 2D), with symmetrical changes in forewing DI elements on both dorsal and ventral surface akin to these of the aberrant female in Figure 1A. The black rings around the hindwing eyespots underwent similarly broad expansions that are not symmetrical in left and right hindwing. In the aberrant male, it is more apparent that, while the ring around the eyespot expanded, the outer black band of hindwing underwent little or no change. A name “Black Eye” is proposed for the eyespot aberrations shown in Figure 1A and Figure 2D. The ventral surface of the wing in “Black Eye” shows considerable expansion and diffusion of the small and compact black ring of control specimens around the small white ventral eyespot that corresponds to the DI element of the dorsal forewing surface ( Figures 1A.ii vs. 1B.ii and Figures 2D.ii vs. 2B.ii). In the “Comet Eye,” the changes in DI element are only noticeable on the ventral surface ( Figure 2A.ii), and it is very likely that the differences in the degree of transformation between the two aberrant males in Figure 2 can be explained by the difference in the dose of heparin they received. Serfas & Carroll (2005) observed no asymmetry in the action of heparin even when it was injected only in one forewing, explaining this observation by suggesting that it influences “the secretion of cold shock hormone by a structure located near the body midline, rather than acting on receptor function within the wing.” While the “Comet Eye” aberrant and the forewing changes in “Black Eye” support this hypothesis, the asymmetry of hindwing pattern changes in male “Black Eye” in Figure 2D, as well as some additional observations provided below, suggest otherwise.

Heparin injections are known to enhance *wingless* gene signaling and have been previously shown to modify forewing DI elements across Lepidoptera ( Martin & Reed, 2014). Özsu *et al.* (2017) recently showed that *wingless* is a regulator of eyespot color patterns in *Bicyclus anynana* butterflies. It is also quite possible, based on the present study, that *wingless* is involved in controlling the black ring around *A. io* eyespot (see reviews of Version 1 of this paper by Martin (2017) and Marcus (2017)). Martin & Reed (2010) proposed that DI elements are found in both forewing and hindwing and that the hindwing ones should “be considered as serial homologs of their forewing counterparts.” Perhaps the black ring of the hindwing dorsal eyespot is homologous with the black ring element of the forewing ventral eyespot as they are positioned very similarly in relation to their respective wing venation ( Supplementary Figure S1 & Supplementary Figure S2), and both react similarly to heparin injections. It is interesting to note that despite the dramatic changes to wing pattern, the ventral hindwing surface remained identical in experimental and control individuals ( Figures 1A.ii vs. 1B.ii; Figures 2D.ii vs. 2E.ii).

Iwata & Otaki (2016), showed correlation between scale size and color in various wing pattern elements of nymphalid butterflies. The close examination of normal dorsal hindwing eyespot reveals that there are at least four different types of scales that form the color pattern in this area: the eyespot center is formed by short, flat scales that are white under normal light and fluorescent under UV; in the blue area of the eyespot, these scales intermix with similarly shaped iridescent blue scales ( Figure 2F.iii and Supplementary Figures). These two types of scales form the eyespot proper and are surrounded by a ring with a top layer of longer, flat, non-iridescent black scales. Beyond the black ring lies yellow or pink wing areas the surface of which are covered with bristle-like scales. In the eyespot transformed by heparin, the expansion of black ring is associated with the expansion of the number of the black-ring type surface scales into the yellow areas of the wing ( Figure 2D.iii). Some of these scales appear separately from the black ring, which can be explained by the intracellular communication between cells within the developing wing membrane via epithelial feet that may be mediating cell rearrangement ( Nardi & Magee-Adams, 1986). Marcus (2017) suggests that observed change may be happening on the level of color pattern determination processes that involves cell-cell signaling by signal transduction processes.

**Figure 4.**
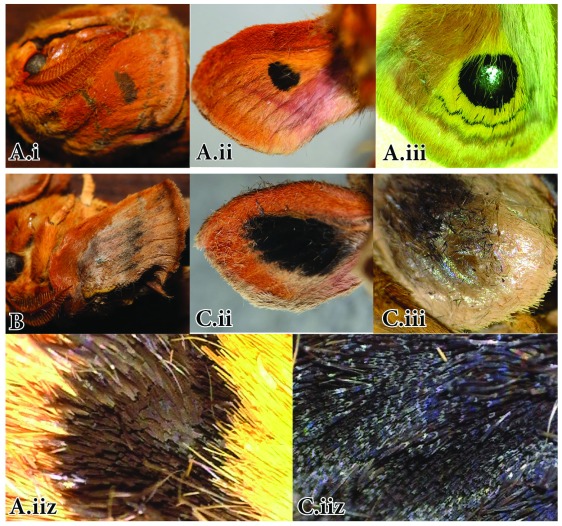
**A–C. Fully-formed *A. io* specimens before wings are expanded** dissected from dead pupae. **A. Control** male; **B,C. Males transformed by heparin** injections (10µl of solution) into pupae ca. 5 hours after pupation. (i) Dorsal forewing surface, (ii) Ventral forewing surface (iiz) Same, zoomed in, (iii) Dorsal hindwing surface. Photos by Andrei Sourakov.

Among the additional five specimens (all males) from dissected dead pupae that were all injected 10µl of heparin solution, the one that was given an injection as a green pupa at about 3 hours after pupation showed no transformation, while four that were injected as brown pupae at ca. five hours after pupation underwent strong transformation that is quite striking even though the wings are not expanded (e. g., Figures 4B,C vs. control specimen in Figure 4A). One of the transformed individuals, shows a higher level of change in the ventral forewing ( Figure 4C.ii) and dorsal hindwing ( Figure 4C.iii). Unlike all other *A. io* modified by heparin injections, the hindwing eyespot is not visible, yielding its place to black scales underlined with scales that appear translucent and produce light diffraction. The dorsal hindwing margin colors and elements are absent in this individual. This level of transformation is comparable to the “Caecus” aberration found in the wild-collected specimen ( Figure 3A). The close examination of the eyespot area in this latter wild specimen ( Figure 3A.i) suggests that it consists of short, flat, iridescent-black-blue scales that differ from long, black scales of the surrounding wing area. In other words, the “Caecus” aberration is the result of the disappearance of the fluorescent white scales and the expansion of the black-ring type scales all the way to the normally yellow hindwing margin.

Such differences in the shape of the scales forming different wing pattern elements is not restricted to the dorsal hindwing. When examining the ventral DI elements of non-expanded forewings more closely in control and transformed moths, shown in Figures 4A.ii and C.ii, it is easy to note that they, too, are formed by differently shaped, flatter, and shorter scales than the surrounding yellow areas that are formed by longer, bristle-shaped scales ( Figures 4A.iiz and 4C.iiz). This also supports the idea that ventral forewing eyespot and dorsal hindwing eyespot, different as they are to the human eye, are homologous organs.

**Figure 5.**
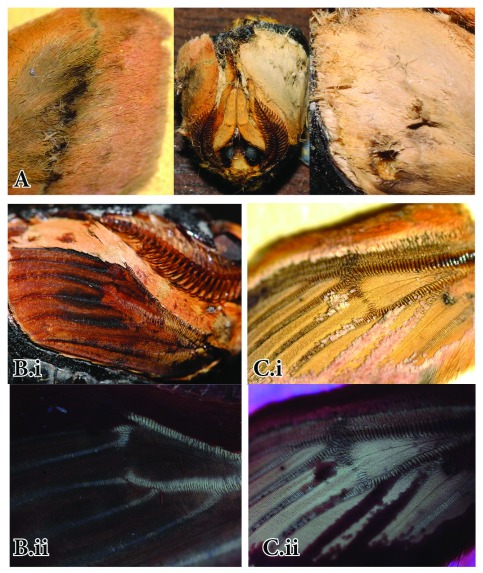
Heparin-induced transformation. **A. Asymmetrically modified forewings** in a male transformed by heparin injections into pupae with (forewing shown in Figure 4C.iii). **B. Forewing membrane**, exposed after pupal case was removed together with scales. **C.** Forewing membrane of control male, scales artificially removed. (i) in LED light, (ii) in UV light. Photos by Andrei Sourakov.

It must be noted that the transformations associated with heparin injections may be more profound than changes in the color and shape of individual scales. In at least one of the four transformed moths (the one whose dorsal hindwing resembles “Caecus” aberration and is illustrated in Figure 4C), the forewings are not symmetrically modified, with little pattern appearing on the right pale forewing ( Figure 5A). Also, during dissections of the dead pupae injected with heparin, the underlying surface of the forewing was revealed ( Figure 5B) and the black pattern ( Figure 5B.i) and change in fluoresce properties ( Figure 5B.ii) as compared to the control ( Figure 5C) suggest that transformation of scales is accompanied by the changes in the underlying wing membrane. Laboratories involved in research on Lepidoptera wing and scale embryology (e. g., Dinwiddie *et al.*, 2014) may find it an interesting avenue of research to pursue.

The wing pattern research has now entered the phase when functions of individual genes are being rapidly revealed (e. g., Marcus, 2005; Monteiro *et al*., 2006; Zhang *et al*., 2017). It is hoped that the present publication, while documenting unique aberrations in a single species, will be useful in the future work directed at understanding wing pattern evolution and development, in general, and will prompt additional experiments that will clarify the observations presented here.

## Supplementary material

**Supplementary File.** Figures S1–S5.

**Figure S1. Forewing venation in *Automeris io* and position of ventral eyespot** in relation to venation. **A. Whole forewing** with pigmentation cleared; **B. Ventral eyespot.** (i) Intact eyespot, photo in LED light; (ii) Scales partially removed to expose venation, photo in UV + incandescent light. Wing venation nomenclature after Heppner, 1998. Photos by Andrei Sourakov

**Figure S2. Hindwing venation in *Automeris io* and position of dorsal eyespot** in relation to venation. **A. Whole forewing** with pigmentation cleared; **B. Dorsal eyespot in** UV + incandescent light. (i) Intact eyespot; (ii) Scales partially removed to expose venation. Wing venation nomenclature after Heppner, 1998. Photos by Andrei Sourakov

**Figure S3. Scales involved in formation** of dorsal hindwing eyespot in *Automeris io* **. A. Dorsal eyespot with some** of the scales removed. **B. Intact eyespot.** Photographed in LED light (i) Whole eyespot; (ii) Close-up of eyespot center; (iii) Close-up of black-ring/yellow field border. (1) white center corresponding to underlying vein, (2) blue part of eyespot, (3) black ring, (4) surrounding yellow field. Photos by Andrei Sourakov

**Figure S4. Three types of scales removed with scotch tape from the** dorsal hindwing eyespot of *Automeris io*. **A. First layer** of scales. **B. Second layer** of scales. Photographed in LED light. **C. Third layer** of scales. Photographed in UV + incandescent light. (1) white center corresponding to underlying vein, (2) blue part of eyespot, (3) black ring, (4) surrounding yellow field. Photos by Andrei Sourakov

**Figure S5. A. Staging of pupae:** time-lapse photographs of a pupa of *Automeris io* reflect the time since pupation. **B–D. Two seven-day-old prepupae and (D) a representative ca. five-hour-old pupa that were injected** with heparin and resulted into transformed moths. (i) and (ii) shedding of larval skin… (iv) 2 hours and 3 minutes after pupation, etc. Photos by Andrei Sourakov & Steven Schlachta.
