# Supplementary material for: Giving eyespots a shiner: Pharmacologic manipulation of the Io moth wing pattern
Source: F1000Res. 2017 Sep 26;6:1319. Originally published 2017 Aug 3. [Version 2] doi: 10.12688/f1000research.12258.2 (PMC5629545; doi:10.12688/f1000research.12258.2)
Supplement: Supplementary file 1 [file f1000research-6-13785-s0000.tgz › d488b6af-b721-40bd-83c9-94170e33c047.pdf]

Supplementary File. Figures S1-S5.
Andrei Sourakov, 2017. *F1000Research*

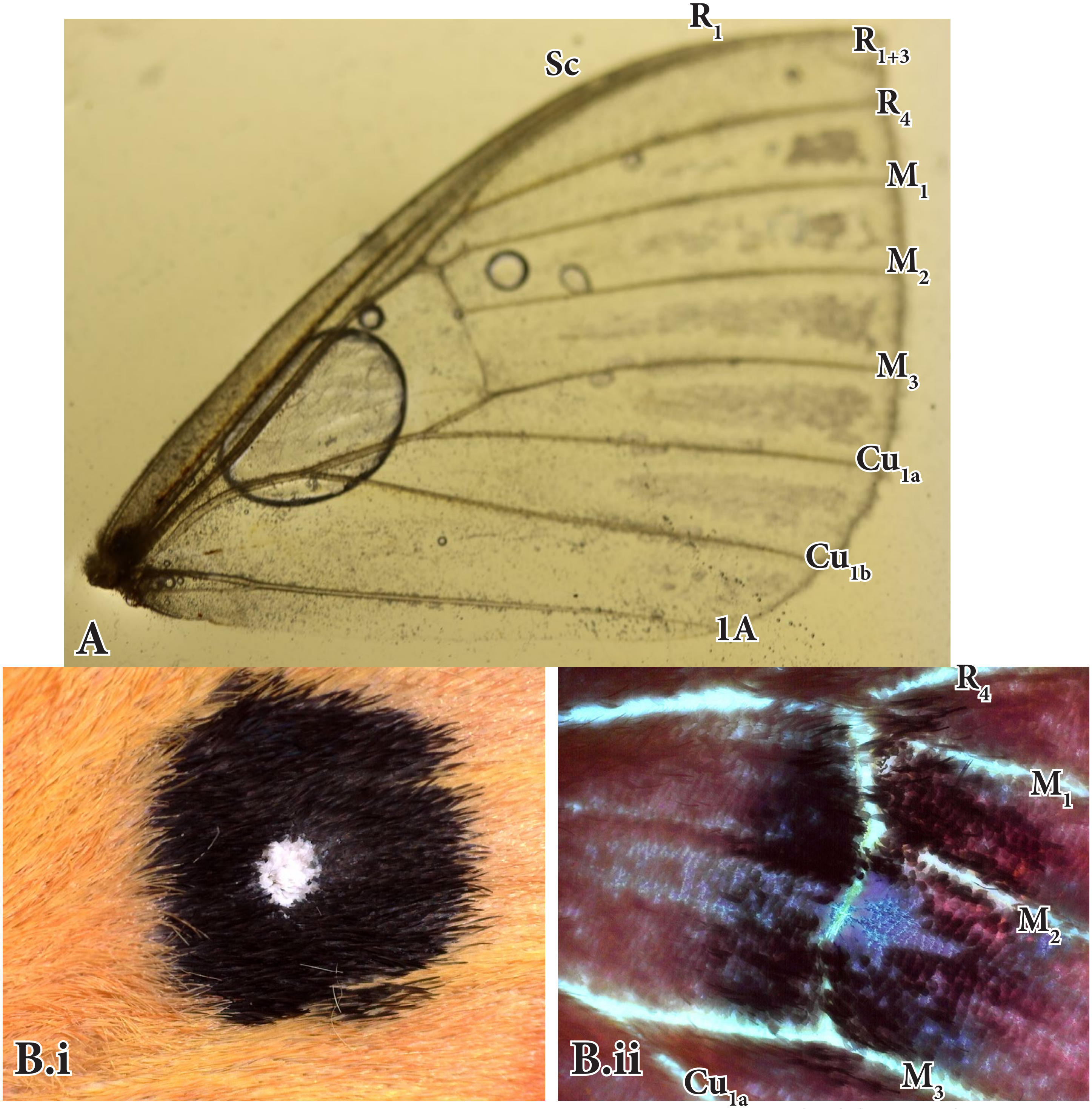

Supplementary Figure S1. Forewing venation in *Automeris io* and position of ventral eyespot in relation to venation. **A. Whole forewing** with pigmentation cleared; **B. Ventral eyespot.** (i) Intact eyespot, photo in LED light; (ii) Scales partially removed to expose venation, photo in UV + incandescent light. Wing venation nomenclature after Heppner, 1998.

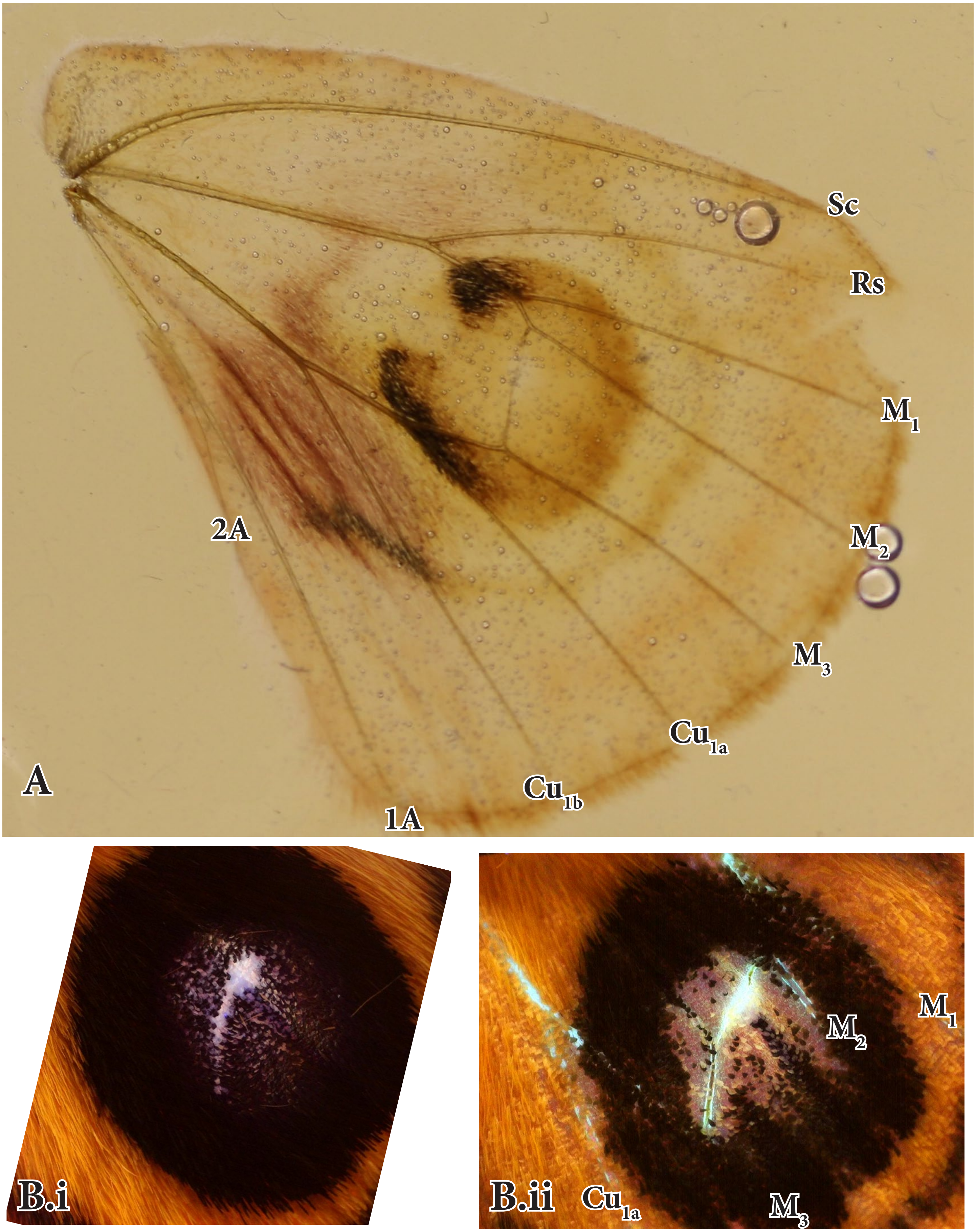

Supplementary Figure S2. Hindwing venation in *Automeris io* and position of dorsal eyespot in relation to venation. **A. Whole forewing** with pigmentation cleared; **B. Dorsal eyespot in** UV + incandescent light. (i) Intact eyespot; (ii) Scales partially removed to expose venation. Wing venation nomenclature after Heppner, 1998.

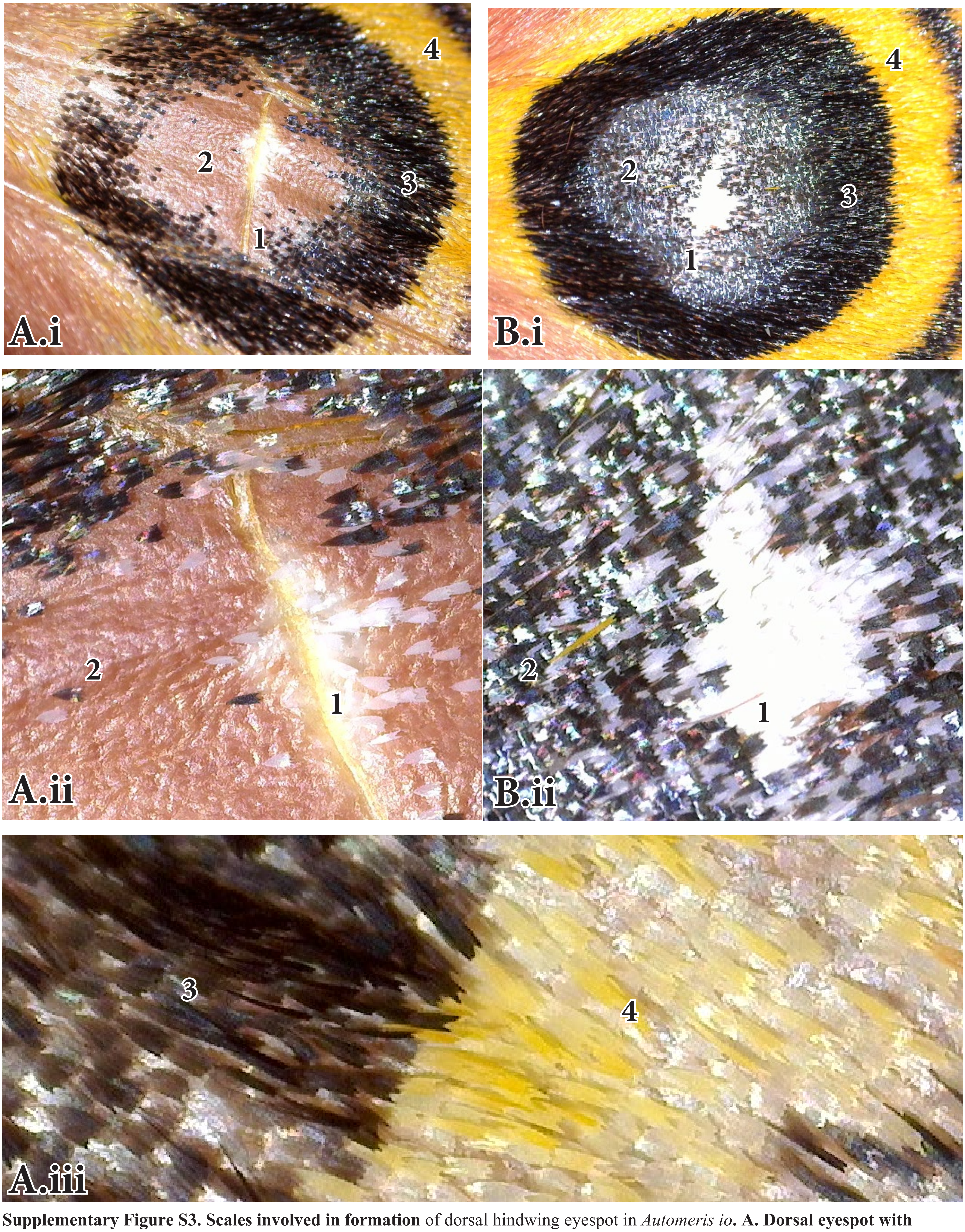

Supplementary Figure S3. Scales involved in formation of dorsal hindwing eyespot in *Automeris io*. **A. Dorsal eyespot with some** of the scales removed. **B. Intact eyespot.** Photographed in LED light (i) Whole eyespot; (ii) Close-up of eyespot center; (iii) Close-up of black-ring/yellow field border. (1) white center corresponding to underlying vein, (2) blue part of eyespot, (3) black ring, (4) surrounding yellow field. Photos by Andrei Sourakov

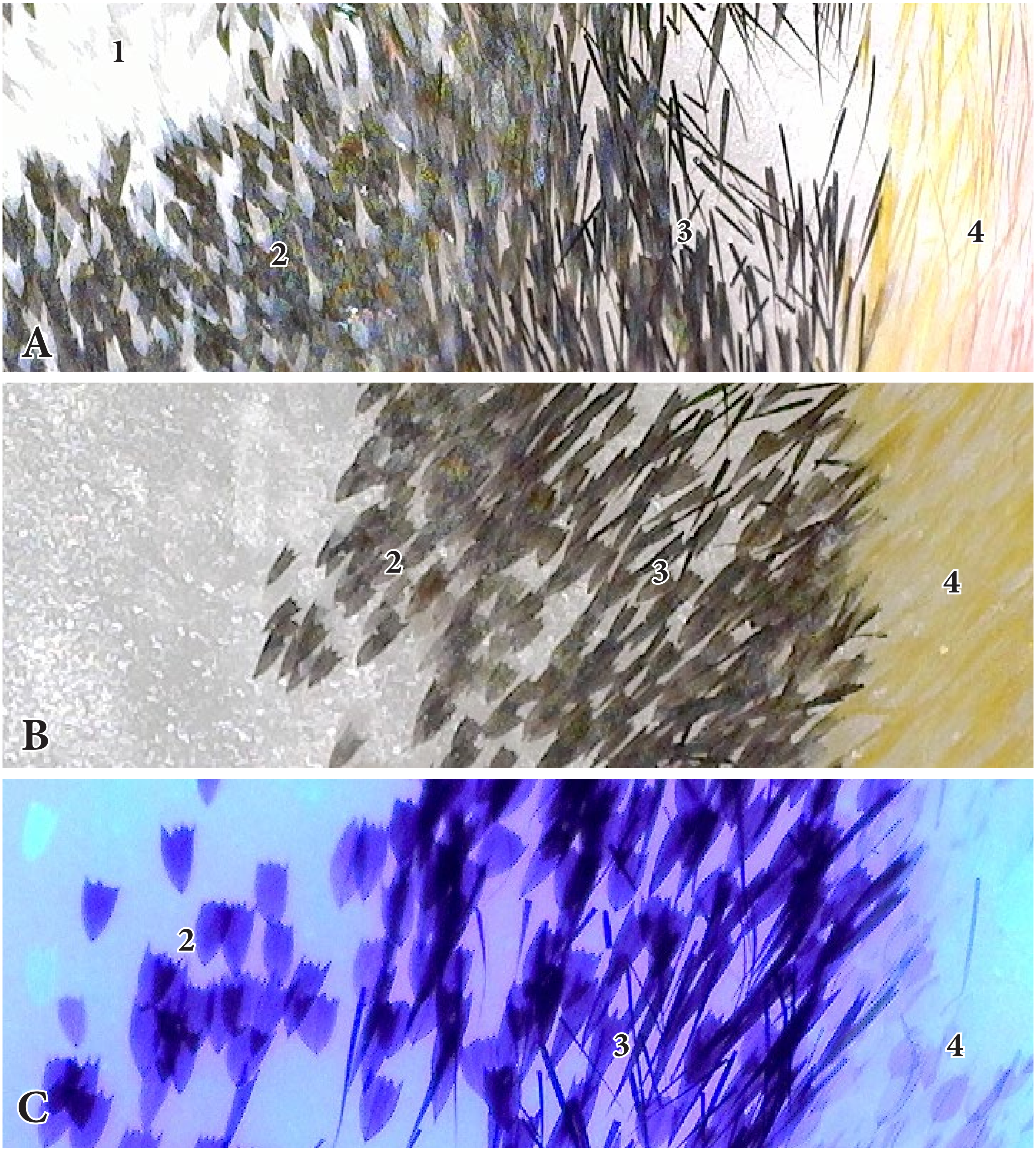

Supplementary Figure S4. Three types of scales removed with scotch tape from the dorsal hindwing eyespot of *Automeris io*. **A. First layer** of scales. **B. Second layer** of scales. Photographed in LED light. **C. Third layer** of scales. Photographed in UV + incandescent light. (1) white center corresponding to underlying vein, (2) blue part of eyespot, (3) black ring, (4) surrounding yellow field. Photos by Andrei Sourakov

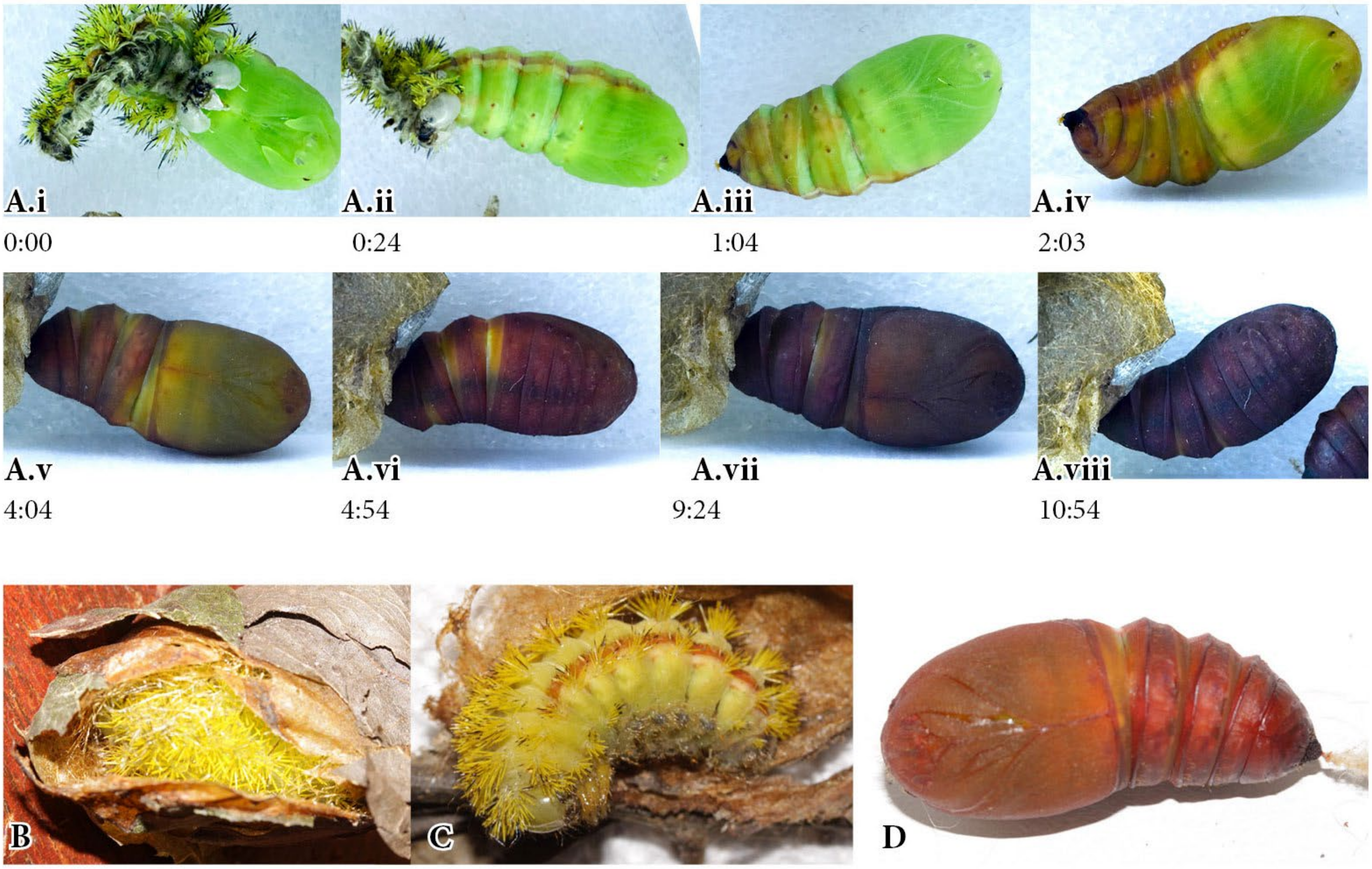

Supplementary Figure S5. A. Staging of pupae: time-lapse photographs of a pupa of *Automeris io* reflect the time since pupation. **B-D. Two seven-day-old prepupae and (D) a representative ca. ive-hour-old pupa that were injected** with heparin and resulted into transformed moths. (i) and (ii) shedding of larval skin… (iv) 2 hours and 3 minutes after pupation, etc.
