# Supplementary material for: Giving eyespots a shiner: Pharmacologic manipulation of the Io moth wing pattern
Source: F1000Res. 2017 Sep 26;6:1319. Originally published 2017 Aug 3. [Version 2] doi: 10.12688/f1000research.12258.2 (PMC5629545; doi:10.12688/f1000research.12258.2)
Supplement: Peer review history

# Peer review: Referee response for version 2

**DOI:** 10.5256/f1000research.13785.r26373

**Published:** 2017-10-04

**Authors:** Arnaud Martin

**Affiliations:** 1 Department of Biological Sciences, The George Washington University, Washington, DC, USA

The author went out his way to improve the first version, and added valuable observational data on replicated experiments. I feel the data is preliminary but useful, and carefully discussed.

I have read this submission. I believe that I have an appropriate level of expertise to confirm that it is of an acceptable scientific standard.

# Peer review: Referee response for version 2

**DOI:** 10.5256/f1000research.13785.r26372

**Published:** 2017-10-02

**Authors:** Jeffrey M. Marcus

**Affiliations:** 1 Department of Biological Sciences, University of Manitoba, Winnipeg, MB, Canada

I congratulate the author for making revisions to create a much improved paper. Thank you for the opportunity to review this work.

I have read this submission. I believe that I have an appropriate level of expertise to confirm that it is of an acceptable scientific standard.

# Peer review: Referee response for version 1

**DOI:** 10.5256/f1000research.13271.r24774

**Published:** 2017-08-17

**Authors:** Jeffrey M. Marcus

**Affiliations:** 1 Department of Biological Sciences, University of Manitoba, Winnipeg, MB, Canada

This manuscript describes a very promising approach to expanding the experimental study of color pattern development beyond the select group of model butterfly species that have received the bulk of the attention thus far. The Saturniid moth *Automeris io* was used for these experimental studies. Also described are *A. io *specimens derived from breeding experiments and wild-caught specimens with aberrant phenotypes.

 While I enjoyed reading the manuscript, there are a number of changes that I would like to suggest to the author:

 Clarification of methods:

 There are a number of methodological details that should be clarified.

1. “sugarberry”. Please give scientific name. Is this *Celtis laevigata*?
2. Staging of pupae. As much detail as possible should be given here about when and how the injections were done relative to the pupal molt. How many hours at what temperature? Was the cuticle sclerotized yet? Where on the experimental specimens’ bodies did the injections take place? Under what conditions were the animals allowed to recover from their injections? What was the manufacturer and purity of the heparin used? For coldshock, how many hours is “overnight”? 
3. Control injections: what was the rationale for using manitol? Many experiments pair heparin with chondroitin sulfate B (keratan sulfate) as a negative control because it is structurally similar to heparin, but lacks the biological activity associated with heparin.
4. Overwintering conditions: the author reports that the experimental animals were kept at ambient conditions until emergence the following spring. Were they kept indoors or outdoors? If indoors what was the typical temperature and lighting conditions during this period? If outdoors, try to provide descriptors of climatic conditions during the appropriate period using National Weather Service or other data.

 

 Clarification of results:

 Additional clarification of the results is also required.

1. The author reports that most of the injected individuals did not emerge. How many failed to emerge? Were there any differences in eclosion rate between the heparin injected individuals and the chondroitin sulfate B injected individuals?
2. What was the emergence rate of unmanipulated individuals? Was it different from the emergence rate of cold-shocked individuals?

 

 Reinterpretation of Results and Elaboration of Discussion:

1. The author should keep in mind that Lepidopteran color patterns can be altered by manipulation of the developmental processes responsible for determining color patterns as well as by manipulation of the developmental processes responsible for color pattern differentiation. Determination processes might involve cell-cell signaling by signal transduction processes, while differentiation of color patterns involves the expression and regulation of biosynthetic pathways responsible for pigment synthesis. Manipulations such as cold-shock might have effects on both kinds of developmental processes, if they are taking place at the time of the manipulation. Additional references to prior work that examines the effects of coldshock (Nijhout 1984; Serfas and Carroll 2005; Mahdi *et al.* 2010; Dhungel and Otaki 2013) might be warranted.
2. Everything that we know about the action of heparin suggests that it interacts with signal transduction pathways such as *wingless*/ *wnt *(Binari *et al.* 1997) *, *and there is no evidence that it interacts with melanin pigment biosynthesis. Discussing the experimental results of heparin injection from this study in reference to what is known about *wingless/wnt* signaling and its effects on color pattern determination in insects (Carroll *et al.* 1994; Monteiro *et al.* 2006; Martin and Reed 2010; Werner *et al.* 2010) would be highly desirable. I also urge the author to extend the discussion further and to connect his work with prior work on Saturniid moth color pattern development including both classic cautery studies (Henke 1933; Henke 1944) and studies of gene expression (Monteiro *et al.* 2006).  Interestingly, the discal eyespots of *Automeris io *are positioned on top of crossveins, structures that also employ *wingless/wnt* signaling during their development (Conley *et al.* 1997; Marcus 2001).  Relating eyespot development to underlying crossvein development in Saturniid moths, perhaps through modulation of the *wingless/wnt* pathway would be a very interesting research trajectory to pursue. Understanding how discal eyespots in some Saturniid moths are both similar to and different from border eyespots in butterflies would be a very interesting avenue for further studies of the evolution and development of Lepidopteran color patterns. 

     

 I would like to encourage the author to consider these points in his revisions and also to continue his experimental explorations in future work.

I have read this submission. I believe that I have an appropriate level of expertise to confirm that it is of an acceptable scientific standard, however I have significant reservations, as outlined above.

## References

[1] BinariRCStaveleyBEJohnsonWAGodavartiRSasisekharanRManoukianAS: Genetic evidence that heparin-like glycosaminoglycans are involved in wingless signaling. *Development*.1997;124(13) :2623-32 921700410.1242/dev.124.13.2623

[2] CarrollSBGatesJKeysDNPaddockSWPanganibanGESelegueJEWilliamsJA: Pattern formation and eyespot determination in butterfly wings. *Science*.1994;265(5168) :109-14 791244910.1126/science.7912449

[3] ConleyCRohwer-NutterDBlairS: Analysis of crossvein patterning in the Drosophila wing using enhancer trap lines and wingless alleles. *Developmental Boilogy*.1997;186(2) :

[4] DhungelBOtakiJ.M: Larval temperature experience determines sensitivity to cold-shock-induced wing color pattern changes in the blue pansy butterfly Junonia orithya. *Journal of Thermal Biology*.2013;38(7) :427-433 Reference source

[5] HenkeK: *Wilhelm Roux Arch Entwickl Mech Org*.1933;128(1) : 10.1007/BF00578945 15-107 10.1007/BF00578945 28354414

[6] HenkeK: Über die Determination der Querbindenzeichnung und die Entstehung der Scheinsymmetrie bei der Saturnide Antheraea pernyi Guer. *Biologisches Zentralblatt*.1944;64:98-148

[7] MahdiSHGimaSTomitaYYamasakiHOtakiJM: Physiological characterization of the cold-shock-induced humoral factor for wing color-pattern changes in butterflies. *J Insect Physiol*.2010;56(9) : 10.1016/j.jinsphys.2010.02.013 1022-31 10.1016/j.jinsphys.2010.02.013 20206631

[8] MarcusJM: The development and evolution of crossveins in insect wings. *J Anat*.199(Pt 1-2) :211-6 1152382510.1046/j.1469-7580.2001.19910211.xPMC1594997

[9] MartinAReedRD: Wingless and aristaless2 define a developmental ground plan for moth and butterfly wing pattern evolution. *Mol Biol Evol*.2010;27(12) : 10.1093/molbev/msq173 2864-78 10.1093/molbev/msq173 20624848

[10] MonteiroAGlaserGStockslagerSGlansdorpNRamosD: Comparative insights into questions of lepidopteran wing pattern homology. *BMC Dev Biol*.2006;6: 10.1186/1471-213X-6-52 52 10.1186/1471-213X-6-52 17090321PMC1654149

[11] NijhoutHF: Colour pattern modification by coldshock in Lepidoptera. *J Embryol Exp Morphol*.1984;81:287-305 6470611

[12] SerfasMSCarrollSB: Pharmacologic approaches to butterfly wing patterning: sulfated polysaccharides mimic or antagonize cold shock and alter the interpretation of gradients of positional information. *Dev Biol*.2005;287(2) : 10.1016/j.ydbio.2005.09.014 416-24 10.1016/j.ydbio.2005.09.014 16216238

[13] WernerTKoshikawaSWilliamsTMCarrollSB: Generation of a novel wing colour pattern by the Wingless morphogen. *Nature*.2010;464(7292) : 10.1038/nature08896 1143-8 10.1038/nature08896 20376004

# Peer review: Referee response for version 1

**DOI:** 10.5256/f1000research.13271.r24773

**Published:** 2017-08-04

**Authors:** Arnaud Martin

**Affiliations:** 1 Department of Biological Sciences, The George Washington University, Washington, DC, USA

This manuscript presents some exciting pattern aberrations observed in the eyespots of the Io Moth, including specimens obtained from drug injections, a wild-caught individual, and previously undescribed collection specimens.

 I enjoyed reading the manuscript and thank the author for publishing this. The reported phenotypes provide valuable information on the formation of eyespot rings in this particularly spectacular species. 

 I noticed the following minor issues.

- What was the rationale for injecting mannitol?
- Serfas and Caroll (2005) -> Carroll with two r (typo)
- Methods: "Ten randomly selected pupae were injected"
  Please provide more details if possible on the staging. Were they tender and relatively fresh? Is there a way to determine the maximum age they were at, or a time estimate based on their cocoon spinning?
  5mg of heparin is a large dose, so it may have killed the younger pupae, while the aberrant female came out by chance by being at a more resilient stage (or was accidentally injected with much less compound)
- Methods, please confirm the injected concentration of the injected heparin (apparently 0.5mg/uL), and provide the exact origin of the compounds (sodium salt? molecular weight? manufacturer?)
- Figure 1 Legend: the abbreviation for DI BR and EIII are missing.
- The author is wrong calling the outer black line "EIII". Schwanwitsch 1956 and Henke 1936 (in 3 other Saturniids) suggest this is MI (central symmetry system outer band). I am personally inclined to say that while it may not be M1 in A. io ... it is certainly not EIII
- "A less aberrant male, whose prepupa was injected with 5mg of heparin a day before pupation, also emerged" 
  I believe the author meant "A less aberrant male, whose prepupa was injected with **2.5mg **of heparin **ONE** day before pupation, also emerged"
- "Heparin injection must have enhanced or prolonged the process of expansion of black pigment once it formed in the black ring around the blue scales."

There is a way to deepen the discussion here, and I will try to explain briefly (feel free to use this suggestion). 

 The A. io Discal Spots (DI = Discalis I element of the Schwanwitsch pattern homology system) are stereotypical patterns that are always overlapping with the discal crossvein. Martin and Reed (2010) suggests that these spots always express the wingless morphogen in Lepidoptera, and Martin and Reed (2014) also shows that DI elements are responsive to heparin treatment in nymphalids. Interestingly, heparin is known to enhance wingless signaling in Drosophila: Baeg *et al. *(2001), Binari *et al* (1997) and Greco *et al* (2001).

 Thus, the eyespot ring expansions observed upon heparin injection here suggest the exciting possibility that wingless, or perhaps other heparin-sensitive morphogens, are deployed during pre-pupal and pupal development to pattern different aspects of the discal ocelli structure. 

 To be honest, the current insight about melanization is a little too phenomenological, because melanin pigment synthesis happens much later in development and there is no known interaction between heparin and melanin biosynthesis... 

I have read this submission. I believe that I have an appropriate level of expertise to confirm that it is of an acceptable scientific standard, however I have significant reservations, as outlined above.

## References

[1] BaegGHLinXKhareNBaumgartnerSPerrimonN: Heparan sulfate proteoglycans are critical for the organization of the extracellular distribution of Wingless. *Development*.2001;128(1) :87-94 1109281410.1242/dev.128.1.87

[2] BinariRCStaveleyBEJohnsonWAGodavartiRSasisekharanRManoukianAS: Genetic evidence that heparin-like glycosaminoglycans are involved in wingless signaling. *Development*.1997;124(13) :2623-32 921700410.1242/dev.124.13.2623

[3] GrecoVHannusMEatonS: Argosomes: a potential vehicle for the spread of morphogens through epithelia. *Cell*.2001;106(5) :633-45 1155151010.1016/s0092-8674(01)00484-6
